# Nucleotides Regulate Secretion of the Inflammatory Chemokine CCL2 from Human Macrophages and Monocytes

**DOI:** 10.1155/2014/293925

**Published:** 2014-09-07

**Authors:** K. R. Higgins, W. Kovacevic, L. Stokes

**Affiliations:** ^1^Sydney Medical School Nepean, University of Sydney, Penrith, NSW 2750, Australia; ^2^Health Innovations Research Institute, School of Medical Sciences, RMIT University, Bundoora, VIC 3083, Australia

## Abstract

CCL2 is an important inflammatory chemokine involved in monocyte recruitment to inflamed tissues. The extracellular nucleotide signalling molecules UTP and ATP acting via the P2Y2 receptor are known to induce CCL2 secretion in macrophages. We confirmed this in the human THP-1 monocytic cell line showing that UTP is as efficient as LPS at inducing CCL2 at early time points (2–6 hours). Expression and calcium mobilisation experiments confirmed the presence of functional P2Y2 receptors on THP-1 cells. UTP stimulation of human peripheral CD14+ monocytes showed low responses to LPS (4-hour stimulation) but a significant increase above background following 6 hours of treatment. The response to UTP in human monocytes was variable and required stimulation >6 hours. With such variability in response we looked for single nucleotide polymorphisms in P2RY2 that could affect the functional response. Sequencing of P2RY2 from THP-1 cells revealed the presence of a single nucleotide polymorphism altering amino acid 312 from arginine to serine (rs3741156). This polymorphism is relatively common at a frequency of 0.276 (*n* = 404 subjects). Finally, we investigated CCL2 secretion in response to LPS or UTP in human macrophages expressing 312Arg-P2Y2 or 312Ser-P2Y2 where only the latter exhibited significant UTP-induced CCL2 secretion (*n* = 5 donors per group).

## 1. Introduction

The CCL2/CCR2 mediated recruitment of monocytes is necessary for fighting infections to microorganisms [1]. This chemokine signalling axis has also been implicated in a number of inflammatory disorders where monocyte infiltration is a key factor such as atherosclerosis, multiple sclerosis, and rheumatoid arthritis [2]. Understanding the cellular regulation of this important chemokine is therefore critical for understanding some of the early pathophysiology of inflammatory disorders.

P2Y receptors are members of the metabotropic family of purinergic receptors belonging to the larger family of G-protein coupled receptors. Eight subtypes of P2Y have been identified to date—P2Y1, P2Y2, P2Y4, P2Y6, P2Y11, P2Y12, P2Y13, and P2Y14—with differences in both pharmacology and downstream signalling pathways [3, 4]. P2Y2 has a widespread distribution in the body including expression on glial cells, some neurons, endothelial cells, epithelial cells of many tissues, and myeloid immune cells including monocytes, macrophages, and dendritic cells [5–10]. Many studies have demonstrated activation of P2Y2 induces a transient calcium response [11, 12] but much less is known about the regulation of chemokine or cytokine production. Our previous work was the first to demonstrate a role for the P2Y2 receptor in regulation of CCL2 secretion from alveolar and peritoneal macrophages [10]. P2Y2 and the UDP-responsive P2Y6 receptor can also signal other chemokine productions including CXCL8 (IL-8) and CCL20 (MIP-3*α*) [9, 13, 14]. Knockout mouse studies have demonstrated that P2Y2 plays an important role in defence against lung infection with* Pseudomonas aeruginosa* [15] yet it can also play a role in allergic lung inflammation in various models [16, 17].

Extracellular nucleotide induction of CCL2 in cells expressing P2Y2 could be an important trigger for the initial recruitment of monocytes to an inflammatory site. Many inducers have been identified for CCL2 including lipopolysaccharide (LPS), growth factors such as platelet derived growth factor (PDGF), and cytokines such as tumour necrosis factor-alpha (TNF-*α*), reviewed in [18]. Following tissue/cellular damage or regulated nucleotide release from cells or nerve terminals, the activation of P2Y2 by extracellular nucleotides could switch on a rapid production of CCL2. Whilst this may be beneficial for initiating repair to an injured site, uncontrolled or chronic nucleotide release may be detrimental and cause excessive tissue inflammation. Both ATP and UTP are known danger signals and act as immunomodulatory signals to communicate alarm messages to immune cells.

The aim of this study was to investigate extracellular nucleotide-induced chemokine production in human macrophages and monocytes with an emphasis on CCL2. Firstly we wanted to confirm our earlier findings in rodent alveolar macrophages using a human monocyte/macrophage cell line and secondly we wanted to compare P2Y2 induced CCL2 secretion with that of LPS, a known bacterial inducer of CCL2. Finally we wanted to perform a pilot study to determine whether we could measure nucleotide-induced CCL2 secretion from primary human cells and assess the responses to both P2Y2 agonists and LPS.

## 2. Materials and Methods

### 2.1. Materials

UTP, ATP, suramin, and LPS (strain 055:B5) were from Sigma-Aldrich (Ryde, Australia). 2-thio-UTP and UTP*γ*S were from Tocris Biosciences (Bristol, UK). Interferon *γ* (IFN-*γ*) was from Roche Diagnostics. MwoI restriction enzyme was from NEB.

### 2.2. Cell Culture

The THP-1 monocytic cell line was maintained in RPMI 1640 media (Life Technologies) containing 10% foetal bovine serum (US origin, Lonza), 2 mM L-glutamine, 100 U/mL penicillin, and 100 *μ*g/mL streptomycin (all Life Technologies) and grown under humidified conditions in a 5% CO_2_ incubator. Cells were routinely passaged every 2-3 days. For differentiation to macrophages, THP-1 cells were plated at 1 × 10^6^ cells/well in 24-well plates in 500 *μ*L complete medium and stimulated with 1000 U/mL IFN-*γ* and 100 ng/mL LPS for 48 hours.

### 2.3. Human Monocyte Preparation

Peripheral venous blood was collected in lithium heparin tubes (Becton Dickinson) from healthy volunteers with informed consent (study approved by Nepean Blue Mountains Local Health Service Human Ethics committee). Mononuclear cells were isolated by Ficoll-Paque (GE Healthcare) gradient centrifugation and monocytes were isolated using CD14 microbeads and LS columns on a midiMACS system (Miltenyi Biotech, Germany) as per manufacturer's instructions. The purity for CD14 monocyte isolations was routinely >90% by flow cytometry.

For macrophage experiments, peripheral blood mononuclear cells were plated in complete RPMI 1640 medium at a density of 2.5 × 10^6^ cells per well. Cells were incubated for 2 hours at 37°C in order to adhere to plastic, nonadherent cells removed, and adherent PBMCs were cultured overnight in 1 mL complete media, washed once the following day, and cultured for 6 more days.

### 2.4. Calcium Measurements

THP-1 cells were harvested from flasks, pelleted at 300 ×g, and resuspended in Fluo-4 NW assay buffer (Life Technologies). THP-1 cells were plated at a density of 2 × 10^5^ cells/well into a 96-well plate coated with poly-D-lysine (Merck Millipore) and were loaded for 30 minutes at 37°C. Human monocytes were plated at 2–4 × 10^5^ cells/well and prepared in the same way. Calcium measurements were performed using a Fluostar OPTIMA plate reader (BMG Labtech) with excitation at 485 nm and emission at 520 nm. All measurements were made at 37°C using a gain setting of 40%.

### 2.5. ELISA Experiments

THP-1 cells were plated at 1 × 10^6^ cells/well in a 24-well plate in RPMI 1640 media containing 1% serum (0.5 mL per well). Stimulations were performed in duplicate. LPS (1 or 10 *μ*g/mL) or nucleotides (varying concentrations) were added directly into the media for 2, 4, 6, or 24 hours. Following stimulation media were removed from the wells into Eppendorfs and centrifuged to remove any contaminating cells. Supernatants were transferred to fresh Eppendorfs and frozen at −80°C. Freshly isolated human peripheral blood CD14^+^ monocytes were plated at 5 × 10^5^ cells/well in RPMI media containing 1% serum. Stimulations were performed in duplicate/triplicate as for THP-1 cells and cell-free supernatants collected after 4 or 6 hours.

Ninety-six well plates (NUNC) were coated with anti-MCP-1 capture antibody (clone 10F7, BD Biosciences) at a concentration of 2 *μ*g/mL in sodium carbonate buffer pH 9.5. Samples and standards (recombinant human MCP-1) were diluted in media. Detection antibody, anti-MCP-1-biotin (clone 5D3-F7, BD Biosciences), was used at 0.5 *μ*g/mL and followed by streptavidin-HRP at 1 *μ*g/mL. TMB-Ultra (PerBioscience) was used for visualisation and 1 M H_2_SO_4_was used as stop solution. Absorbance at 450 nm was read using a BMG Labtech Optima plate reader. Standard curves were fit with regression factor of *r* ^2^ > 0.96.

### 2.6. Real-Time PCR

Cells were stimulated as described above, collected into RNA Protect reagent (Qiagen), and stored at −80°C. Cells were then processed to extract total RNA using an RNEasy Mini Kit (Qiagen). RNA concentrations were measured using a spectrophotometer (Cary) using absorbance at 260 nm. 1 *μ*g RNA was reverse transcribed using a Tetro cDNA synthesis kit (Bioline) as per manufacturer's instructions.

Primers for real-time PCR were optimised for concentrations over the range (0.125 *μ*M to 1 *μ*M) and primer efficiencies were determined. Primer sequences were *β*-actin forward 5′-GCC CTG GCA CCC AGC ACA AT-3′ and reverse 5′-GGA GGG GCC GGA CTC GTC AT-3′, GAPDH forward 5′-CGA GAT CCC TCC AAA ATC AA-3′ and reverse 5′-TTC ACA CCC ATG ACG AAC AT-3′, CCL2 forward 5′-CCC CAG TCA CCT GCT GTT AT-3′ and reverse 5′-GAG TTT GGG TTT GCT TGT CC-3′, CCL20 forward 5′-AAG TTG TCT GTG TGC GCA AAT CC-3′ and reverse 5′-CCA TTC CAG AAA AGC CAC AGT TTT-3′, CCL3 forward 5′-ACT TTG AGA CGA GCA GCC AGTG-3′ and reverse 5′-TTT CTG GAC CCA CTC CTC ACTG-3′, and CXCL8 forward 5′-GAG AGT GAT TGA GAG TGG ACC AC-3′ and reverse 5′-CAC AAC CCT CTG CAC CCA GTT T-3′. Quantitative real-time PCR was performed using Sensimix SYBR green No ROX mastermix (Bioline) and 0.75 *μ*M each primer on freshly prepared cDNA. PCR were performed in triplicate using a Rotorgene 2000 (Corbett Research) and analysed using a threshold of 0.003 to determine Ct values. Data was analysed using the 2^−ΔΔCt^ method.

### 2.7. Flow Cytometry

One million THP-1 or peripheral blood mononuclear cells were stained per flow tube. Cells were fixed with 2% paraformaldehyde buffer for 20 minutes on ice and permeabilised with 0.1% saponin in PBS. Rabbit IgG was used as the negative control and anti-P2Y2 (Sigma) was used at 1 : 100 dilution. Primary antibodies were incubated for 30 minutes in PBS/0.1% saponin containing 5% human AB serum. Cells were co-stained with mouse anti-human CD14-FITC (1 : 100 dilution). PBMCs were washed with PBS/saponin and incubated with goat anti-rabbit IgG-Alexa 647 at 1 : 100 dilution for 30 minutes on ice. After final washing with PBS/saponin, cells were resuspended in PBS and 30000 events acquired on a BD FACSCalibur flow cytometer. Monocytes were identified by CD14 expression and plots of P2Y2-Alexa-647 staining were generated using Weasel flow cytometry software (WEHI).

### 2.8. Genotyping

Genomic DNA was prepared from whole blood as previously described [19] and stored at −80°C. The P2Y2 gene was amplified using 0.025 U/mL recombinant* Taq* DNA polymerase (Invitrogen), 1.5 mM MgCl_2_, 100 *μ*M dNTP, and 0.4 *μ*M each of forward and reverse primers. Primer sequences were forward 5′-CTT TTG CCG TCA TCC TTG TCT-3′ and reverse 5′-CAT CTC GGG CAA AGC GTA-3′ yielding a product of 328 bp. The following cycling conditions were used: initial denaturation (95°C for 3 minutes), followed by 40 cycles of denaturation (95°C for 45 seconds), annealing (55°C for 30 seconds), and extension (72°C for 30 seconds) on a PTC-200 Peltier Thermal Cycler (MJ Research, Waltham, Massachusetts, USA). The samples were then cooled at 4°C for 10 minutes.

A restriction assay was designed to determine the sequence at nucleotide 1269, G, or C. Mutation from G>C introduces an extra cut-site for the restriction enzyme MwoI. P2Y2 PCR product was incubated with 3 U MwoI for 1 hour at 60°C. Genomic DNA carrying G at nucleotide 1269 will be cut at 2 positions yielding 3 fragments of 195 bp, 130 bp, and 3 bp. Genomic DNA carrying C at nucleotide 1269 will be cut at 3 positions yielding 4 fragments of 181 bp, 130 bp, 14 bp, and 3 bp. The assay distinguishes between bands of 195 bp and 181 bp using a 3% agarose gel. Genotyping was verified using a commercial high-throughput method assay for rs3741156 (AGRF, Brisbane).

### 2.9. Statistical Analysis

Data plotted are means ± SEM of three to four experiments. Graphs and statistical analysis were performed using GraphPad Prism version 5 (GraphPad Software Inc., La Jolla, CA, USA).

## 3. Results

### 3.1. P2Y2 Regulates CCL2 Production in THP-1 Monocytes

**Figure 1:**
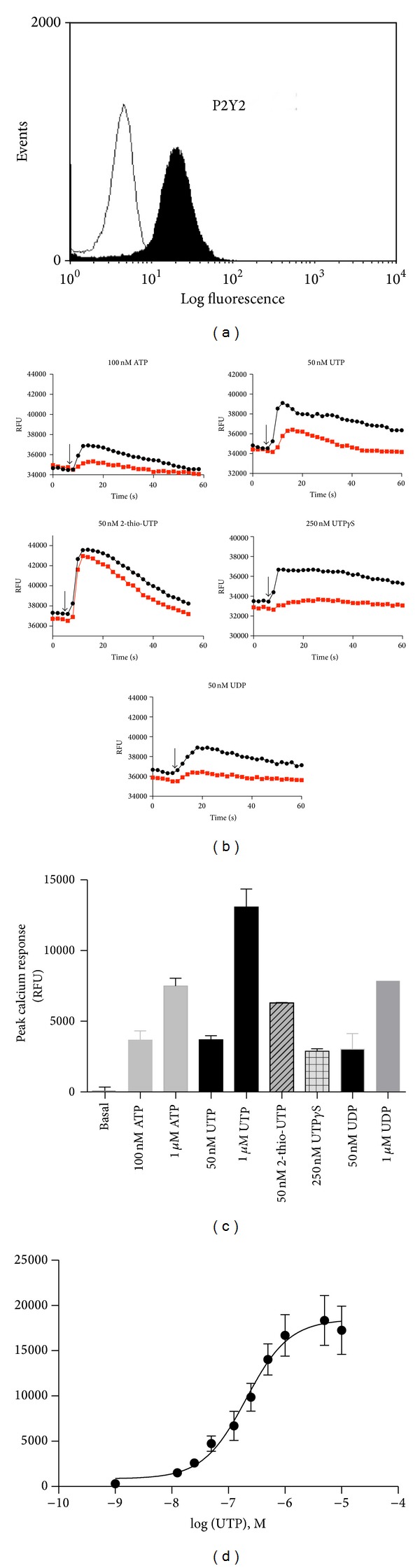
Nucleotides induce intracellular calcium responses in THP-1 cells. (a) Representative plot showing P2Y2 expression on THP-1 monocytes using a specific anti-P2Y2 antibody and a BD FACSCalibur flow cytometer. (b) THP-1 cells were loaded with Fluo-4 using a NW kit and plated at 2 × 10^5^ cells/well in poly-D-lysine coated 96-well plates. Intracellular calcium responses were measured using a BMG Labtech Fluostar Optima plate reader for over 60 seconds at 37°C. Responses to 100 nM ATP, 50 nM UTP, 50 nM 2-thio-UTP, and 250 nM UTP*γ*S are shown in black. Paired responses of cells pretreated with the P2 receptor antagonist suramin (30 *μ*M; 30 minutes) are shown in red. (c) Mean calcium response data from several experiments. (d) A concentration-response curve was generated for UTP-induced calcium responses on THP-1 cells. Mean responses ± SEM are plotted from 4 separate experiments.

It has been demonstrated by others that THP-1 cells express P2Y2 receptors [13, 20]. We confirmed P2Y2 expression on THP-1 using flow cytometry (Figure 1(a)) and used a functional measure of P2Y2 receptors by intracellular calcium measurements in response to a range of P2Y2 nucleotide agonists, ATP, UTP, 2-thio-UTP, UTP*γ*S, and UDP (Figures 1(b) and 1(c)). A concentration-response curve was generated for UTP-induced calcium responses on THP-1 cells (Figure 1(d)). Calcium responses were reduced in the presence of suramin (100 *μ*M), a broad P2 receptor antagonist known to block P2Y2 responses (Figure 1(b)).

**Figure 2:**
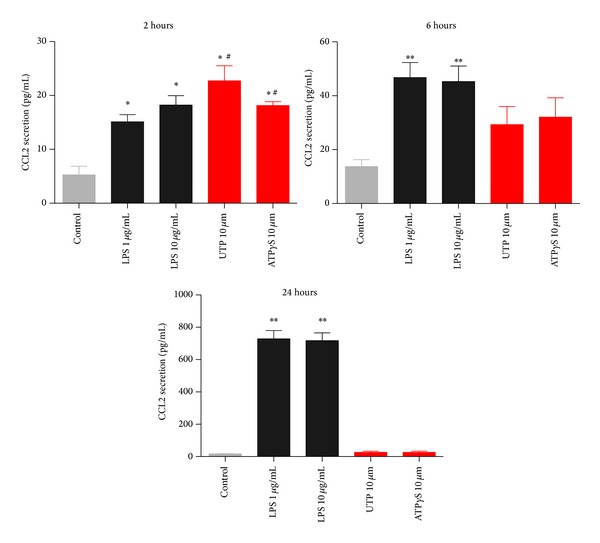
The effect of P2Y2 agonists compared with LPS on CCL2 secretion from unprimed THP-1 cells. THP-1 cells were plated in RPMI media containing 1% serum and stimulated with media alone, LPS (1 or 10 *μ*g/mL), UTP (10 *μ*M), or ATP*γ*S (10 *μ*M) for the times indicated above each graph. Mean raw data is plotted in pg/mL ± SEM from three independent experiments. Symbols: ∗ denotes *P* < 0.05 compared with control, # denotes no significant difference between treatments, and ns denotes not significant with respect to control (one-way ANOVA with Tukey's post hoc test). Standard curves were performed for each ELISA experiment with fits of *r* > 0.95.

**Figure 3:**
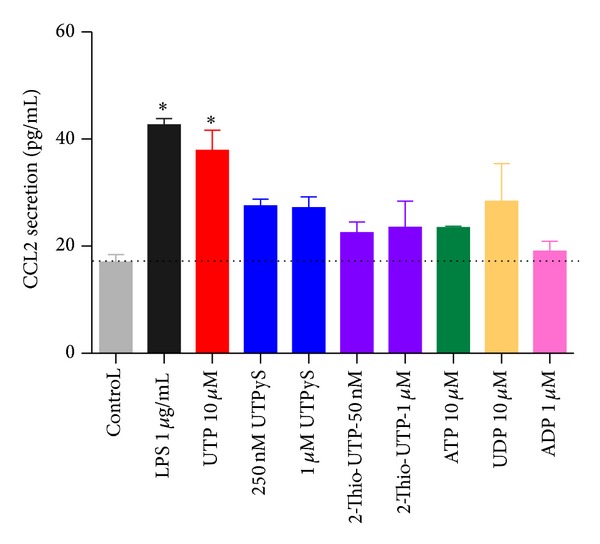
Nucleotides induce CCL2 secretion from THP-1 cells after 2-hour stimulation. THP-1 cells were plated in RPMI media containing 1% serum and stimulated with media alone, LPS (1 *μ*g/mL), UTP (10 *μ*M), UTP*γ*S (250 nM and 1 *μ*M), 2-thio-UTP (50 nM and 1 *μ*M), ATP (10 *μ*M), UDP (10 *μ*M), or ADP (10 *μ*M) for 2 hours. Mean raw data is plotted in pg/mL ± SEM. Standard curves were performed for each ELISA experiment with fits of *r* > 0.95.

**Figure 4:**
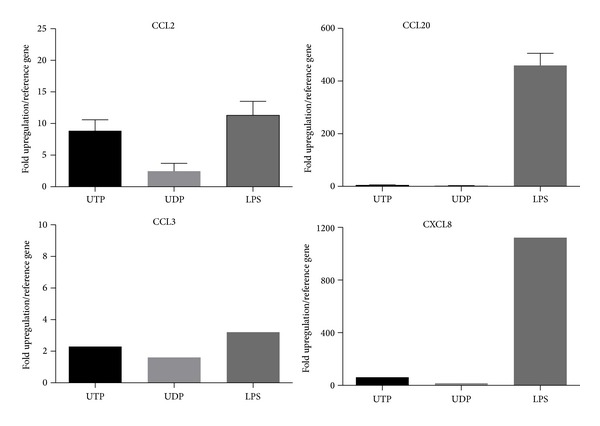
UTP induces CCL2 gene transcription in THP-1 monocytes. THP-1 cells were plated in RPMI media containing 1% serum and stimulated with media alone, LPS (1 *μ*g/mL), UTP (10 *μ*M), or UDP (10 *μ*M) for 2 hours. RNA was extracted, reverse transcribed to cDNA, and probed with quantitative PCR primers for chemokines (CCL2, CCL20, CCL3, and CXCL8) and reference genes (*β*-actin and GAPDH). PCR was performed in triplicate using a Rotorgene 2000. Data was analysed using the 2^−ΔΔCt^ method using a threshold of 0.003.

We then stimulated THP-1 cells with the nucleotides UTP and ATP*γ*S to stimulate P2Y2, or with LPS (1–10 *μ*g/mL) to induce CCL2 chemokine production and secretion. We determined the amount of CCL2 secreted at three separate timepoints: 2 hours, 6 hours, and 24 hours (Figure 2). We found that UTP and ATP*γ*S were as effective as LPS at stimulating CCL2 secretion after 2 hours. After 6 hours the UTP or ATP*γ*S-induced CCL2 secretion remained elevated above basal and again was not significantly different to LPS. However, after 24 hours of stimulation there was a further increase in the LPS-induced CCL2 secretion, while the UTP or ATP*γ*S-induced CCL2 secretion remained low (Figure 2). We decided to study the 2-hour timepoint to further investigate nucleotide-induced CCL2 secretion. A range of different nucleotide agonists were tested and many increased CCL2 levels above background levels (Figure 3). However, we found that only UTP or LPS treatments gave a significant difference compared to basal (one-way ANOVA with Dunnett's post hoc test, *n* = 3-4 experiments). The UTP signal was not blocked by suramin, a feature also observed when concentrations of UTP higher than 100 nM were used in calcium responses in THP-1 cells (data not shown). Using the THP-1 cell line we looked at CCL2 gene induction by UTP in comparison to LPS. We used quantitative real-time PCR and measured CCL2 relative to *β*-actin as the reference gene at 2 hours following stimulation with LPS, UTP, UDP, or media alone. We found that UTP induced an 8.8-fold increase in CCL2 expression compared to an 11-fold upregulation of CCL2 in response to LPS (Figure 4). In contrast UDP did not induce a significant upregulation of CCL2 levels (Figure 4). As expected, LPS induced other chemokines CCL20 (459-fold above media control) and CXCL8 (1121-fold above media control) in THP-1 cells, whereas UTP treatment induced a 4.1-fold upregulation of CCL20 and a 2.3-fold upregulation of CCL3 with respect to unstimulated cells. UTP induced some upregulation of CXCL8 expression (61-fold) but this was only 5% of the LPS response (1121-fold).

### 3.2. CCL2 Production in CD14^+^ Primary Human Monocytes

**Figure 5:**
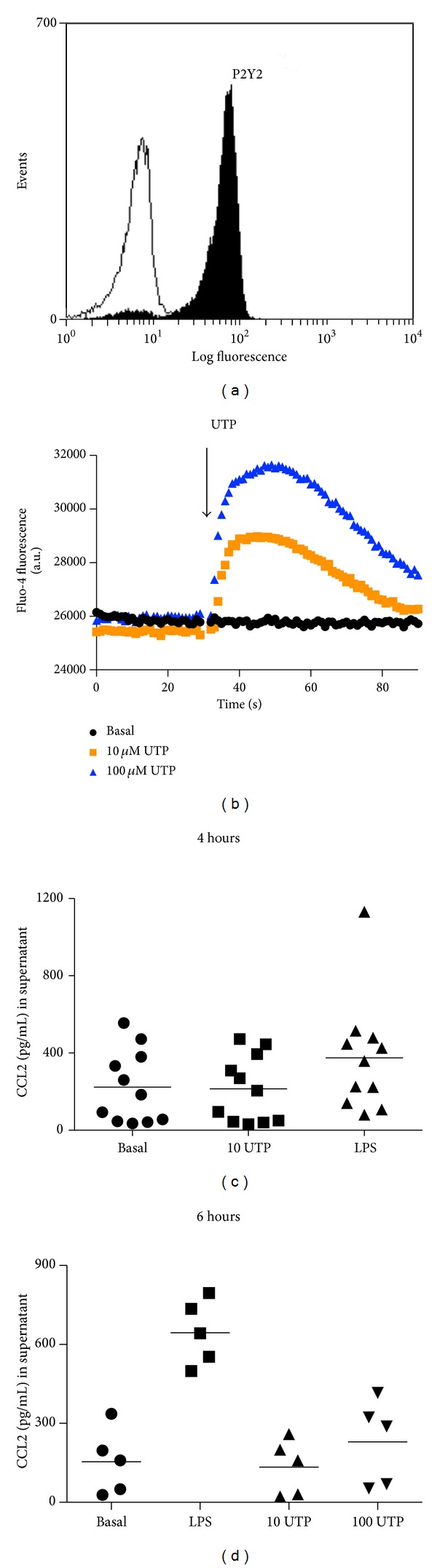
Primary human peripheral blood monocytes express P2Y2 but display variable CCL2 secretion to UTP. (a) Human PBMCs were labelled with conjugated CD14-FITC and rabbit anti-P2Y2 followed by goat anti-rabbit Alexa 647 using an intracellular staining protocol. (b) Human CD14 positive magnetically isolated monocytes were plated on poly-D-lysine plates and loaded with Fluo-4AM. UTP induced a transient calcium response at both 10 *μ*M (orange) and 100 *μ*M (blue). Calcium measurements were recorded using a Fluostar Optima plate reader at 37°C. (c) Human CD14^+^ monocytes were plated at 5 × 10^5^ cells/well and stimulated with media, LPS 100 ng/mL, or 10 *μ*M UTP for 4 hours. Each symbol represents a different donor (*n* = 11 donors in total). (d) Human CD14^+^ monocytes stimulated for 6 hours with media, LPS 100 ng/mL, 10 *μ*M UTP, or 100 *μ*M UTP. Each symbol represents a different donor (*n* = 5 different donors).

Following our observations that UTP could induce a similar level of CCL2 secretion as LPS in the THP-1 monocytic cell line, we wanted to address whether this was also a feature of primary human monocytes. We probed CD14-positive PBMCs for P2Y2 expression and found significant labelling relative to a nonspecific IgG control (Figure 5(a)). To assess the functionality of P2Y2 receptors on magnetically isolated CD14 positive monocytes we performed calcium measurements on Fluo-4AM loaded cells. UTP at both 10 *μ*M and 100 *μ*M induced large transient calcium responses in primary monocytes (Figure 5(b)).

We stimulated human CD14^+^ monocytes with media, UTP (10 *μ*M), or LPS (100 ng/mL) for 4 hours (*n* = 11 different donors). When analysing the data as collective, we found a small increase in CCL2 in response to LPS (mean 376 ± 88 pg/mL) above background CCL2 secretion in media alone (mean 223 ± 56 pg/mL). There was no difference above background with 10 *μ*M UTP treatment (mean 215 ± 51 pg/mL) (Figure 5(c)). When looking at each individual donor we could see that in 4 out of 11 subjects UTP increased CCL2 levels above media alone treated cells. Subtracting the background CCL2 concentration showed a UTP response with a mean of 23 pg/mL (*n* = 4 subjects, range 8–49 pg/mL).

We then determined whether a longer incubation time of 6 hours would reveal an increased CCL2 secretory response to UTP. When analysing the data as a collective, we found a significant increase in CCL2 in response to LPS (mean 645 ± 55 pg/mL, *n* = 5 donors, *P* < 0.05 one-way ANOVA with Dunnett's post hoc test) compared to background (mean 154 ± 55 pg/mL, *n* = 5 donors). Treatment with 10 *μ*M UTP (mean 134 ± 47 pg/mL) or 100 *μ*M UTP (229 ± 72 pg/mL, *n* = 5 donors) was not significantly different from background CCL2 levels (Figure 5(d)). Again, when looking at each subject we could see that all 5 donors responded to 100 *μ*M UTP with CCL2 levels ranging from 19 to 129 pg/mL above the basal secretion with a mean value of 76 pg/mL.

**Figure 6:**
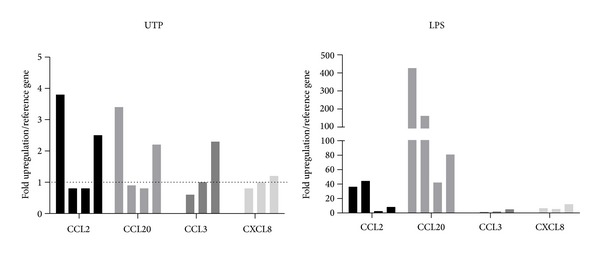
UTP induces chemokine gene transcription in human monocytes. Human CD14^+^ monocytes were plated in RPMI media containing 1% serum and stimulated with media alone, LPS (1 *μ*g/mL), UTP (10 *μ*M), or UDP (10 *μ*M) for 6 hours. RNA was extracted, reverse transcribed to cDNA, and probed with quantitative PCR primers for chemokines (CCL2, CCL20, CCL3, and CXCL8) and reference gene (*β*-actin). PCR was performed in triplicate using a Rotorgene 2000. Data was analysed using the 2^−ΔΔCt^ method using a threshold of 0.003 and fold upregulation with respect to the media control is plotted.

To determine whether we could measure CCL2 gene induction in primary human monocytes we performed qPCR analysis of CCL2 gene induction by UTP (100 *μ*M) and LPS after a 6-hour stimulation. We found variable induction of CCL2 and CCL20 in response to either UTP or LPS (Figure 6, *n* = 3-4 donors).

### 3.3. Does P2Y2 Play a Role in CCL2 Secretion in Human Macrophages?

**Figure 7:**
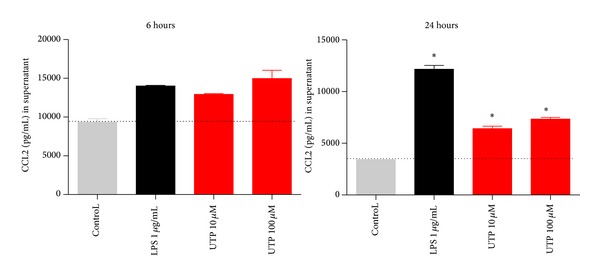
IFN/LPS differentiated THP-1 macrophages display increased CCL2 production to LPS or UTP. THP-1 cells were plated in RPMI media containing 10% serum and stimulated with IFN-*γ* and LPS for 48 hours. Media were removed and cells were challenged with either media alone (control), LPS (1 *μ*g/mL), UTP (10 *μ*M), or UTP (100 *μ*M) for 4 hours. Mean raw data is plotted in pg/mL ± SEM. Standard curves were performed for each ELISA experiment with fits of *r* > 0.95.

With such small responses to UTP in monocytes and unstimulated THP-1 monocytic cells, we decided to differentiate THP-1 to macrophage-like cells using IFN-*γ* and LPS [20]. Our previous work was performed using an alveolar macrophage cell line and peritoneal macrophages and measured robust CCL2 production [10]. IFN-*γ*/LPS differentiated THP-1 cells (48-hour treatment) were stimulated with either LPS, UTP, or media control and CCL2 was measured in supernatants collected at 6 and 24 hours. A much higher basal secretion of CCL2 was measured compared to unstimulated THP-1 but both LPS and UTP could further increase CCL2 secretion at both timepoints (Figure 7).

**Figure 8:**
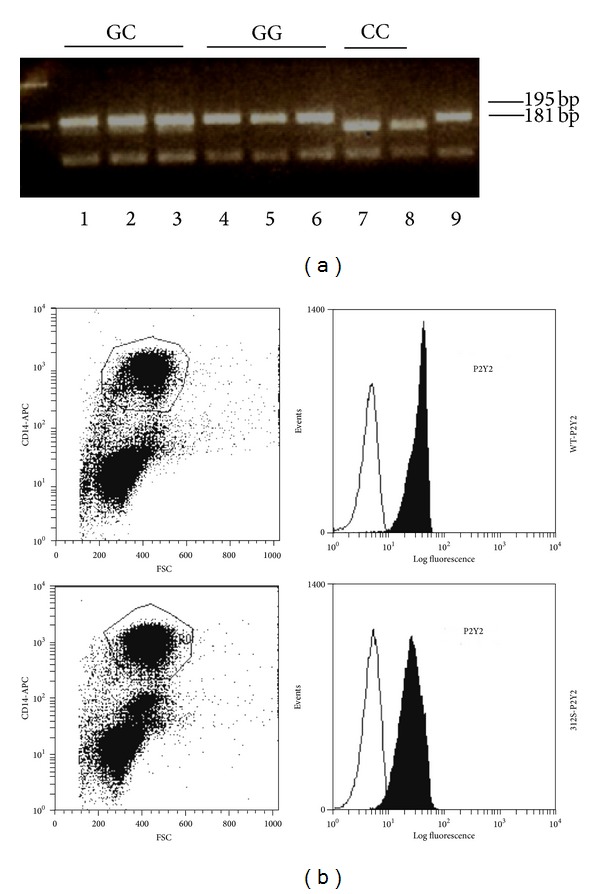
P2Y2 expression is similar in macrophages expressing 312Arg-P2Y2 and 312Ser-P2Y2 variants. (a) An in-house genotyping assay was designed using PCR and restriction enzyme analysis with MwoI. Individuals with GG (wild-type, lanes 4, 5, and 6) have a single upper band of 195 bp. Individuals with CC (homozygous for 312Ser, lanes 7 and 8) show a single upper band of 181 bp. Individuals with GC genotype (heterozygous for 312Ser, lanes 1, 2, and 3) show two upper bands at 195 and 181 bp. (b) Flow cytometry dot plots showing gating of CD14 positive PBMCs and representative histograms showing P2Y2 expression on CD14^+^ gated population.

To begin to address the variability in responses observed with primary monocytes we focused on nonsynonymous single nucleotide polymorphisms in P2Y2. There are three known SNPs in human P2Y2 which may affect functional responses [21, 22]. We first sequenced the P2Y2 gene from THP-1 monocytes and found several synonymous SNPs and a single nonsynonymous SNP (rs3741156) altering amino acid 312 from arginine (R) to serine (S) (data not shown). We developed an in-house genotyping assay based on restriction enzyme analysis with MwoI. The presence of G or C at position 1269 (NM_002564) correlates with a cut-site for this enzyme. Wild-type individuals carrying G at this position would show 3 fragments of 195, 130, and 3 bp, whereas polymorphic individuals carrying C at this position would show 4 fragments in the PCR restriction assay of 181, 130, 14, and 3 bp (Figure 8(a)). We used this assay to genotype P2Y2 in healthy volunteers and confirmed the genotyping using a custom high-throughput SNP assay (Australian Genome Research Facility). From genotyping a total of 404 subjects we determined an allele frequency of 0.276 for rs3741156 (*n* = 404 subjects).

**Figure 9:**
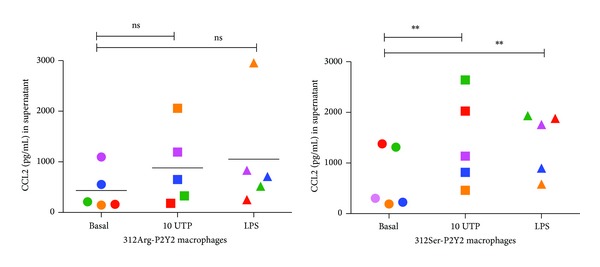
Macrophages expressing 312Ser-P2Y2 secrete higher levels of CCL2 in response to UTP and LPS. Human adherent PBMCs were plated at 2.5 × 10^6^ cells/well and cultured for 7 days. Media were replaced and cells were stimulated with media, 10 *μ*M UTP, or 100 *μ*g/mL LPS for 4 hours. Each symbol represents a different donor (*n* = 5 donors per genotype group) and each donor has a different colour. Bars represent the mean data for each condition. ∗∗ denotes *P* < 0.05 using a one-way ANOVA with Dunnett's post hoc test; ns denotes not significant.

We then performed a pilot study to investigate the effect of genotype on chemokine secretion using individuals carrying wild-type (WT) P2Y2 or 312S-P2Y2 receptors (5 subjects per genotype). We stained PBMCs from WT and 312S-P2Y2 subjects for expression level of P2Y2 on CD14 positive cells and found no significant difference in mean fluorescence intensity (*n* = 3 donors/genotype) (Figure 8(b)). We cultured adherent PBMCs to macrophages over 7 days to differentiate monocytes to macrophages and stimulated cells with media, 10 *μ*M UTP, or 1 *μ*g/mL LPS for 4 hours (*n* = 5 donors per genotype group). Supernatants were tested for CCL2 by ELISA and results are shown in Figure 9 as a scatter graph. Responses to both LPS (mean 1054 ± 485 pg/mL) and UTP (mean 882 ± 341 pg/mL) were smaller in WT-P2Y2 macrophages and not significantly different from background CCL2 (mean 433 ± 181 pg/mL, *n* = 5 donors). In contrast, responses to LPS (mean 1411 ± 280 pg/mL) and UTP (mean 1416 ± 401 pg/mL) were significantly different from background CCL2 (mean 682 ± 271 pg/mL, *n* = 5 donors, one-way ANOVA with Dunnett's post hoc test) in 312S-P2Y2 expressing macrophages.

## 4. Discussion

This study is the first to investigate UTP induced chemokine secretion from THP-1 cells and primary human monocytes and macrophages. Our main finding suggests that UTP can elicit comparable levels of CCL2 as stimulation with the bacterial product LPS in THP-1 cells. We also found that macrophages produce more CCL2 in response to nucleotides than monocytes even though levels of P2Y2 expression are similar.

It is well known in the literature that monocytes and macrophages express P2Y2 receptors amongst other P2Y and P2X receptors [3, 4, 11, 13]. Here we confirm expression of P2Y2 in THP-1 cells using flow cytometry and intracellular calcium measurements as an indirect measure of G protein-coupled receptor activation (Figure 1). The pharmacology for this receptor is relatively poor compared with other purinergic receptors. Several pieces of evidence suggest that the UTP-induced response in THP-1 cells is due to P2Y2 activation. UTP is only known to activate P2Y2, P2Y4, and P2Y6 receptors [23], and, of these, the only receptor responsive to ATP is P2Y2. ATP and UTP are equipotent at P2Y2 receptors and EC_50_ values for UTP and ATP-induced calcium responses were 97 ± 34 nM and 105 ± 59 nM, respectively, in THP-1 cells. Suramin could suppress UTP and ATP-induced calcium responses as well as the response elicited by UTP*γ*S (Figure 1). However, suramin did not suppress responses induced by the P2Y2-selective agonist 2-thio-UTP (Figure 1). Furthermore, we found that suramin could not suppress calcium responses induced by high concentrations (>1 *μ*M) of nucleotides and as such we did not use suramin in the subsequent CCL2 experiments.

The calcium experiments were used as an indication that functional P2Y receptors were present on THP-1 cells and we found that the P2Y6 receptor agonist UDP also elicited a suramin-sensitive calcium response in THP-1 cells (Figure 1). Whilst P2Y6 is likely expressed in THP-1 cells as shown by Yebdri et al. [13], experiments with CCL2 secretion suggest that UDP did not have a significant our effect (Figure 3). Furthermore, both UTP*γ*S (P2Y2 and P2Y4 selective agonist) and 2-thio-UTP (P2Y2 selective) could induce CCL2 secretion above basal levels but not to the same degree as UTP (full agonist at P2Y2).

A major aim of this study was to compare nucleotide-induced chemokine secretion to a known proinflammatory signal such as LPS. We chose the THP-1 cell line to perform these experiments to limit variability in responses. The LPS-induced CCL2 secretory response in THP-1 monocytes was low (<100 pg/mL) at early timepoints such as 2 and 6 hours but increased over a 24-hour treatment period (Figure 2). The amount of constitutively produced CCL2 from THP-1 cells in our hands was similar to that measured by Steube et al. [24]. Steube et al. observed a low level of CCL2 secretion from THP-1 in response to LPS compared to other myelomonocytic cells lines [24]. This low constitutive CCL2 secretion is quite different from the high level of constitutive CCL2 expression seen in NR8383 alveolar macrophages in our previous study [10].

We compared the LPS-induced CCL2 response to that of UTP and ATP*γ*S at concentrations known to induce large calcium responses (10 *μ*M) in THP-1 cells. At an early timepoint, 2 hours, the nucleotide-induced CCL2 secretion was comparable to LPS-induced CCL2 secretion (Figure 2). However, the nucleotide-induced response remained stable between 2 and 6 hours and the LPS-induced response steadily increased over time. In primary human monocytes and macrophages the kinetics of CCL2 production appeared to be slower in response to either LPS or UTP (Figures 5 and 9). After 4–6 hours of stimulation the LPS-induced CCL2 response becomes significant above background CCL2 secretion, whereas overall the UTP-induced response remains not significantly elevated above basal levels (Figure 5). Thus in primary human monocytes we observed a large difference between the P2Y2-induced response and the LPS-induced response. Several factors may influence this data including genetic differences in membrane receptors, expression levels of receptors at the plasma membrane, and differences in intracellular signalling or in CCL2 mRNA stability. However, in human monocyte-derived macrophages the UTP-induced response was again similar to the LPS-induced response (Figure 9) and this was also true in IFN/LPS differentiated THP-1 macrophages after 6 hours (Figure 7).

We investigated whether nucleotides could also induce production of other inflammatory chemokines such as CCL20 (MIP-3*α*), CCL3 (MIP-1*α*), and CXCL8 (IL-8). Nucleotide-induced CCL20 production has been demonstrated in human dendritic cells (UDP and ATP*γ*S) [9], CXCL8 production in monocytes [13], and CCL3 production in rodent microglia (UTP, UDP) [7]. Using quantitative PCR we found that LPS upregulated both CCL20 and CXCL8 in THP-1 cells; however UTP had no significant effect on CCL3, CCL20, and CXCL8 production (Figure 4). In primary human monocytes LPS significantly induced CCL20 gene expression in all 4 donors (42–427-fold increase). In comparison UTP and UDP nucleotides did not significantly induce CCL20 (*n* = 4 donors). Our data shows that CCL2 induction in primary human CD14^+^ monocytes was variable in response to either LPS or UTP (Figure 6) confirming our variability in the protein secretion experiments.

The finding that UTP stimulation of P2Y2 receptors can elicit a similar CCL2 chemokine response to LPS stimulation of TLR4 is a novel finding and one with potential relevance for inflammation. UTP is a known danger signal, similar to ATP, and is likely to be present in areas of tissue damage without infection in addition to sites of infection. Such nucleotides may be a driving factor in sterile inflammation and may contribute to disease associated chronic inflammatory states. It is also important to hypothesise about why nucleotides may induce CCL2. In addition to the role of CCL2 as a chemokine for immune cells, it may be released as a mechanism for upregulating other receptors such as P2X4 receptors, as recently described by Toyomitsu et al. [25]. CCL2 may therefore act as an autocrine factor on monocytes/macrophages to prime further inflammatory signalling.

A second major aim of the current study was to investigate P2Y2 induced chemokine production in primary human monocytes and macrophages. We isolated monocytes from peripheral blood of a number of donors to determine if the nucleotide-induced CCL2 response was detectable. Our data demonstrates a degree of variability in constitutive and induced secretion of CCL2 from monocytes and macrophages. This type of variability has been observed previously for LPS where different individuals can be classified as high or low responders [26] and other studies have demonstrated variable LPS-induced responses including interleukin 1*β* (IL-1*β*) secretion [27] and CCL20 chemokine secretion from human dendritic cells and monocytes [9]. We and others have also previously shown variability in IL-1*β* secretion in response to P2X7 activation [19, 28]. One source of such variability in responses is genetic variation in the form of single nucleotide polymorphisms and three SNPs have been identified in the human* P2RY2* gene [21, 22]. Sequencing determined that one SNP, rs3741156, was present in the THP-1 cell line and other studies have demonstrated that this mutation altering amino acid 312 can affect UTP-induced calcium responses in transfected cells [21]. Other studies have indicated that P2Y induced calcium signalling is important in switching on CCL2 production in myeloid cells [7]. Monocyte-derived macrophages from individuals carrying the 312Ser-P2Y2 variant displayed a significant CCL2 secretory response compared to individuals expressing a 312Arg-containing receptor (Figure 9). Therefore, SNPs in P2Y2 may correlate with variability in responses. Future studies will investigate if other SNPs are present in the gene and whether they have a functional effect on receptor signalling.

## 5. Conclusions

Nucleotides are effective inducers of the chemokine CCL2 from THP-1 monocytic cell line similar to lipopolysaccharide. Human primary macrophages display a more robust CCL2 response to nucleotides than human monocytes. Some of the variability in the CCL2 response to nucleotides could be explained by genetic variation in the P2Y2 gene with a mutation altering amino acid 312 demonstrating an increased chemokine response.
